# Oblique angle deposition of nickel thin films by high-power impulse magnetron sputtering

**DOI:** 10.3762/bjnano.10.186

**Published:** 2019-09-20

**Authors:** Hamidreza Hajihoseini, Movaffaq Kateb, Snorri Þorgeir Ingvarsson, Jon Tomas Gudmundsson

**Affiliations:** 1 Science Institute, University of Iceland, Dunhaga 3, IS-107 Reykjavik, Iceland; 2 Department of Space and Plasma Physics, School of Electrical Engineering and Computer Science, KTH Royal Institute of Technology, SE-100 44, Stockholm, Sweden

**Keywords:** glancing angle deposition (GLAD), high-power impulse magnetron sputtering (HiPIMS), oblique angle deposition, magnetron sputtering, magnetic anisotropy, nickel

## Abstract

**Background:** Oblique angle deposition is known for yielding the growth of columnar grains that are tilted in the direction of the deposition flux. Using this technique combined with high-power impulse magnetron sputtering (HiPIMS) can induce unique properties in ferromagnetic thin films. Earlier we have explored the properties of polycrystalline and epitaxially deposited permalloy thin films deposited under 35° tilt using HiPIMS and compared it with films deposited by dc magnetron sputtering (dcMS). The films prepared by HiPIMS present lower anisotropy and coercivity fields than films deposited with dcMS. For the epitaxial films dcMS deposition gives biaxial anisotropy while HiPIMS deposition gives a well-defined uniaxial anisotropy.

**Results:** We report on the deposition of 50 nm polycrystalline nickel thin films by dcMS and HiPIMS while the tilt angle with respect to the substrate normal is varied from 0° to 70°. The HiPIMS-deposited films are always denser, with a smoother surface and are magnetically softer than the dcMS-deposited films under the same deposition conditions. The obliquely deposited HiPIMS films are significantly more uniform in terms of thickness. Cross-sectional SEM images reveal that the dcMS-deposited film under 70° tilt angle consists of well-defined inclined nanocolumnar grains while grains of HiPIMS-deposited films are smaller and less tilted. Both deposition methods result in in-plane isotropic magnetic behavior at small tilt angles while larger tilt angles result in uniaxial magnetic anisotropy. The transition tilt angle varies with deposition method and is measured around 35° for dcMS and 60° for HiPIMS.

**Conclusion:** Due to the high discharge current and high ionized flux fraction, the HiPIMS process can suppress the inclined columnar growth induced by oblique angle deposition. Thus, the ferromagnetic thin films obliquely deposited by HiPIMS deposition exhibit different magnetic properties than dcMS-deposited films. The results demonstrate the potential of the HiPIMS process to tailor the material properties for some important technological applications in addition to the ability to fill high aspect ratio trenches and coating on cutting tools with complex geometries.

## Introduction

The realization of electronics based on utilizing the electron spin degree of freedom, commonly referred to as spintronics, requires the integration of ferromagnetic films with semiconductors [1]. Nickel is a ferromagnetic heavy 3d transition metal that crystallizes in the fcc structure. Because of the negative magnetostriction property of pure nickel, it is used as a magnetic material for certain applications, including ones that utilize magnetostriction. Thin nickel films have also found a wide range of other applications such as decorative coatings [2–3], corrosion-resistant coatings [3–4], optically transparent conductive electrodes [5], contact devices [6], Li-storage materials [7], and as selective absorbers in solar thermal energy conversion [8]. Moreover, a number of nickel-containing alloys exploit the ferromagnetic properties of nickel such as NiTi-based shape memory alloy thin films utilized in micro-actuator applications [9]. It is well known that microstructure, texture and structure of thin films can have significant influence on the magnetic and other functional properties of the films. The magnetic properties of evaporated [10–11], electrodeposited [12–15], chemical-vapor-deposited [16], and dc [17–19] and rf [20–22] magnetron sputtered thin nickel films have been studied for almost ten decades. This has included studies of the magnetic properties while varying film thickness [10,20], grain size, substrate material [11,21] and substrate temperature [19], as well as while stacking into superlattices [23–24].

High-power impulse magnetron sputtering (HiPIMS), sometimes referred to as high-power pulsed magnetron sputtering (HPPMS), is a physical vapor deposition (PVD) technique based on pulsed power technology where the peak power exceeds the time-averaged power by roughly two orders of magnitude [25–26]. By pulsing the cathode target to high peak power density a high ionization fraction of the sputtered material is achieved, which results in a higher quality of the deposited films [27]. It is well known that ferromagnetic materials are difficult to sputter with conventional dc magnetron sputtering since a portion of the magnetic flux is shunted by the magnetic target, thus decreasing the electron confinement, which results in low plasma density and low deposition rate. On the other hand, it has been demonstrated that a small decrease in the magnetic field strength in the HiPIMS process can lead to a significant increase in the deposition rate in that case [28–29]. We have recently reported an increase by a factor of 2 and 2.6 of the HiPIMS deposition rate by 83% and 53% weakening of the magnetic field strength (at racetrack) using vanadium [30] and titanium [31] targets, respectively. Thus, utilizing HiPIMS for the deposition of ferromagnetic material can be very beneficial.

Oblique deposition, sometimes referred to as glancing angle deposition (GLAD), is known as a PVD technique that leads to a film texture with low density and columnar grain growth that is elongated in the direction of the incoming flux [32]. As a result of this structure, some unique optical [33–35], electrical [36–37], mechanical [37–38] and magnetic [39] properties of thin films have been reported. By employing an ionized deposition flux (i.e., using HiPIMS in GLAD), the angular distribution of the deposited material can be influenced [40–42]. Earlier we have explored the microstructure and magnetic properties of polycrystalline [43] and epitaxially [44] deposited permalloy (Ni_80_Fe_20_ atom %) thin films deposited under 35° tilt using dcMS and HiPIMS. The films prepared by HiPIMS present a lower anisotropy field (*H*_k_) and coercivity (*H*_c_) than films deposited with dcMS. For the polycrystalline films both deposition methods give uniaxial magnetic anisotropy due to the oblique deposition. However, for the epitaxial films dcMS deposition gives biaxial anisotropy while HiPIMS deposition gives a well-defined uniaxial anisotropy. The uniaxial anisotropy induced by the tilt angle was demonstrated in the early 1960s by Smith et al. [39] while depositing permalloy with thermal evaporation. They suggested that a shadow effect causes an in-plane texture perpendicular to the direction of the incoming flux, which corresponds to the easy axis of the film. However, more recently there are reports on a 90° rotation of the easy axis in a Co film deposited at 75° tilt angle [45].

In the present study we investigate the effect of angle of incidence on the structural and magnetic properties of Ni thin films deposited using dcMS and HiPIMS. We chose to work with pure Ni rather than NiFe alloys because it rejects many proposed explanations for uniaxial anisotropy based on alloying, i.e., directional ordering of Fe/Ni atom pairs [46], shape anisotropy of an elongated ordered phase [47], composition variation between grains [48] and, more recently suggested, localized composition non-uniformity [49]. Besides, we do not rotate the substrate during the deposition to simplify the conditions at the cost of losing film thickness uniformity.

## Experimental

The nickel thin films were deposited in a custom-built magnetron sputter chamber [50] with a base pressure of 4 × 10^−6^ Pa. For the deposition process, 32 sccm of argon of 99.999% purity was injected into the chamber as the working gas. The working gas pressure was kept at 0.6 Pa using a butterfly valve located between the chamber and a turbomolecular pump. The nickel target was 75 mm in diameter, of 99.95% purity, and 1.59 mm thick but almost 40% eroded at the racetrack center. The magnetic field measured at the target surface over the racetrack shows the value of 39 and 0 mT parallel and perpendicular to the target surface, respectively.

For HiPIMS operation the power was supplied by a SPIK1000A pulse unit (Melec GmbH) operating in the unipolar negative mode at constant voltage, which in turn was fed by a dc power supply (ADL GS30). The discharge current and voltage were monitored using a combined current transformer and a voltage divider unit (Melec GmbH) and the data were recorded with a custom-made LabVIEW program. The pulse length was set at 200 μs and the pulse repetition frequency was kept at 100 Hz throughout this study. For dcMS operation, a dc power supply (MDX 1 K, Advanced Energy) was connected to the magnetron. For all films, depositions were made at 150 W average power. This corresponds to a peak current density of *J*_D_*_,_*_peak_ = 0.77 A/cm^2^ for the HiPIMS deposition process when averaged over the entire target area. HiPIMS and dcMS oblique angle depositions were made at substrate tilt angles of 0° (substrate faces the target), 35° and 70° using both deposition methods. In addition, more depositions under 10° and 20° by dcMS and 50° and 60° using HiPIMS were made for better understanding of the magnetic properties of the films. The distance between target and substrate position was 25 cm. We used thermally oxidized Si(001) with an oxide thickness of 100 nm as substrates. However, for the scanning electron microscopy studies, Si(001) substrates with native oxide were used in order to eliminate the charging effect. All films were deposited at room temperature (25 °C) with a grounded substrate holder.

X-ray diffractometry (XRD) was carried out using a Philips X’pert diffractometer (Cu Kα, wavelength 0.15406 nm) mounted with a hybrid monochromator/mirror on the incident side and a 0.27° collimator on the diffraction side. A line focus was used with a beam width of approximately 1 mm. The grazing incidence (GI)XRD scans were carried out with the incident beam at θ = 1°. Average thickness (*d*_ave_), average surface roughness and mass density of the films were determined by low-angle X-ray reflectivity (XRR) measurements with an angular resolution of 0.005°, and the data was fitted using the Parrat formalism [51]. A low-density surface layer (around 1 nm) on top of the film had to be included in the model in order to achieve a good fit. This is due to the formation of an oxide or oxynitride surface layer after the films were removed from the vacuum chamber, as has been previously observed and discussed [52]. However, the reported mass density values are corresponding to the “bulk” part of the film.

The film thickness gradient (Δ*d*) was characterized by non-contact mode atomic force microscopy (AFM) analysis in an XE-100 multi-mode AFM system (PSIA Inc.) in air (ex situ). For this aim, the edges of the substrate were marked before deposition. After deposition, the samples were sonicated in an ethanol/isopropanol mixture to remove the marker and the nickel on top of it (lift-off process).

Cross sections of the Ni films were studied using a Leo Supra 25 scanning electron microscope. The acceleration voltage of the electron beam was set to 20 kV and the working distance was kept at 3.5 mm for all images presented here.

Magnetic hysteresis was characterized using a custom-made high-sensitivity magneto-optical Kerr effect (MOKE) looper using a laser source with 632.8 nm wavelength. Coercivity was read directly from the easy-axis loops. In our uniaxial samples the anisotropy field is obtained by extrapolating the linear low-field trace along the hard-axis direction to the saturation magnetization level, a method commonly used when dealing with effective easy-axis anisotropy. Vibrating sample magnetometry (VSM) was performed on 10 × 10 mm^2^ sized samples at 300 K. Variable magnetic fields up to ±1 T were used for magnetic measurements.

## Results and Discussion

### Thin film structure

**Figure 1:**
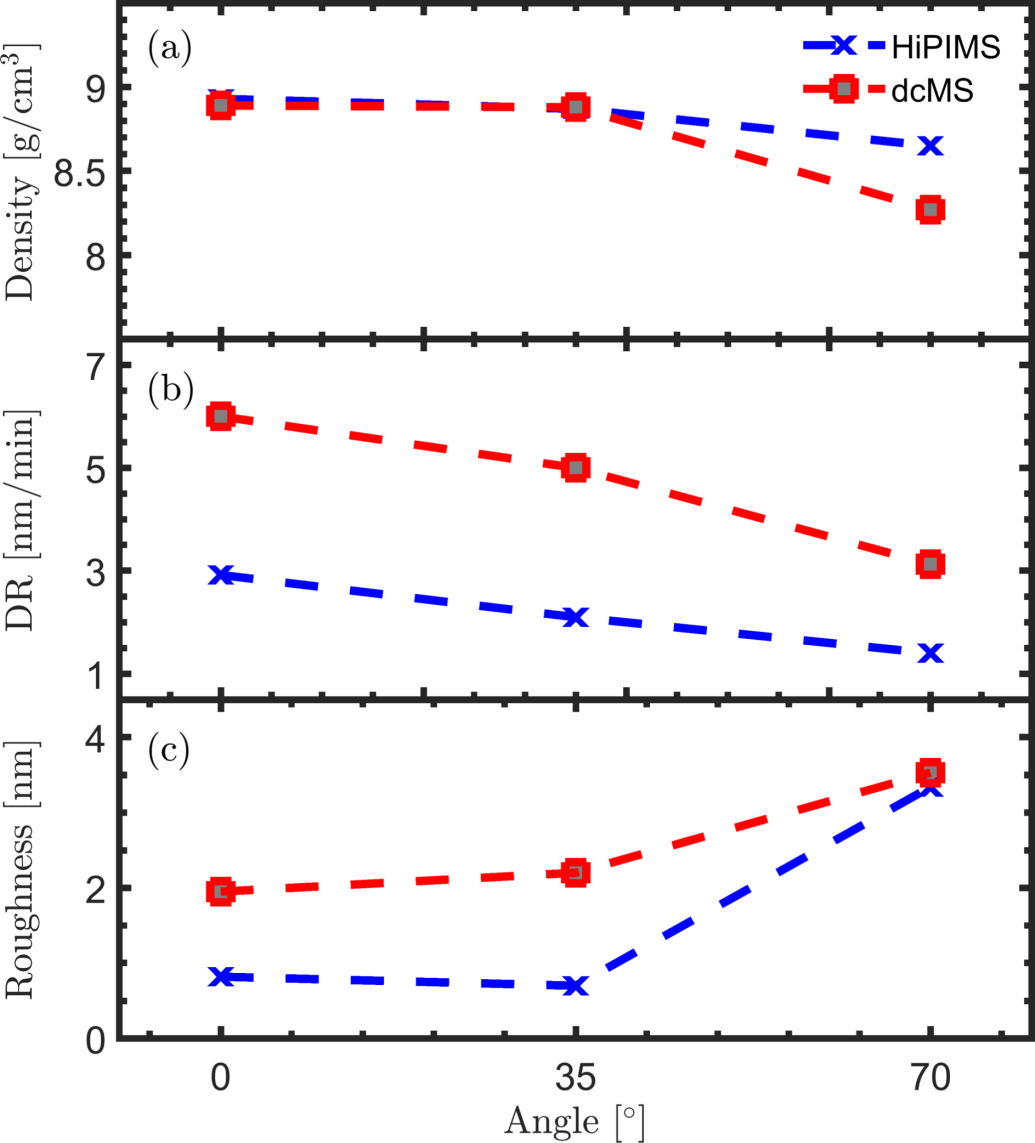
(a) Film density, (b) deposition rate, and (c) surface roughness of nickel films deposited by HiPIMS and dcMS. The data was extracted from XRR measurements. All films are deposited at 0.6 Pa working gas pressure, 150 W average power. In the HiPIMS case we used a pulse length of 200 μs and a repetition frequency of 100 Hz.

**Figure 2:**
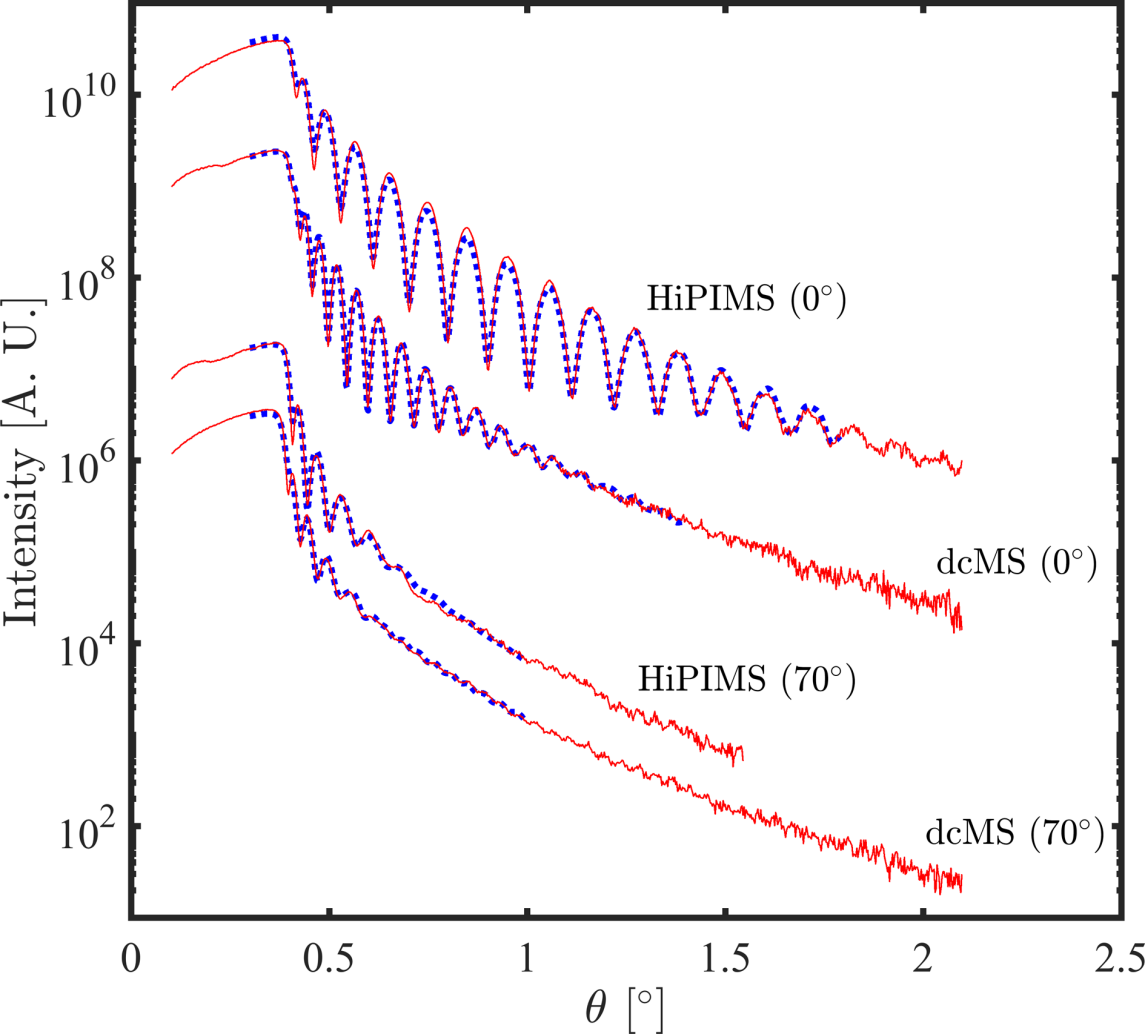
The measured (red solid) and simulated (blue dot) XRR data of HiPIMS and dcMS deposited Ni films under 0- and 70-degrees tilt angles.

Figure 1 shows the film density, deposition rate and surface roughness of Ni films deposited by HiPIMS and dcMS at tilt angles of 0°, 35° and 70 °. Both methods result in similar film densities at 0° and 35° (8.90 and 8.87 g/cm^3^ respectively). The bulk density of nickel at room temperature is 8.902 g/cm^3^[3]. Increasing the tilt angle to 70° leads to a drop in density for both deposition methods. Data extracted from XRR shows density values of 8.6 g/cm^3^ for the HiPIMS-deposited and 8.27 g/cm^3^ for the dcMS-deposited film. We calculated the average deposition rate by dividing the average thickness by the deposition time, and it is shown in Figure 1b for each tilt angle. Deposition rates of 2.92, 2.10 and 1.41 nm/min were calculated for HiPIMS deposition at 0°, 35° and 70°, respectively. The dcMS deposition rate is roughly two times that of the HiPIMS rate for the same tilt angles. This is a somewhat lower deposition rate than has been reported for rf magnetron sputtering of Ni in the past [20–21], which might be due to rather long distance between target and substrate (25 cm) in this experiment. In terms of surface roughness, the HiPIMS-deposited film shows 0.8 nm roughness while the dcMS-deposited film shows 1.9 nm for normal deposition. The surface roughness remains unchanged for deposition at 35°, for both methods. Increasing the tilt angle to 70° leads to a significant change in the surface roughness of the HiPIMS-deposited film (3.3 nm), which is slightly smoother than the dcMS-deposited film (3.5 nm). Note that due to the thickness gradient, fitting the XRR data for films deposited at higher tilt angles includes greater uncertainty. The measured and simulated XRR data are presented in Figure 2 for depositions under tilt angles of 0° and 70°.

**Figure 3:**
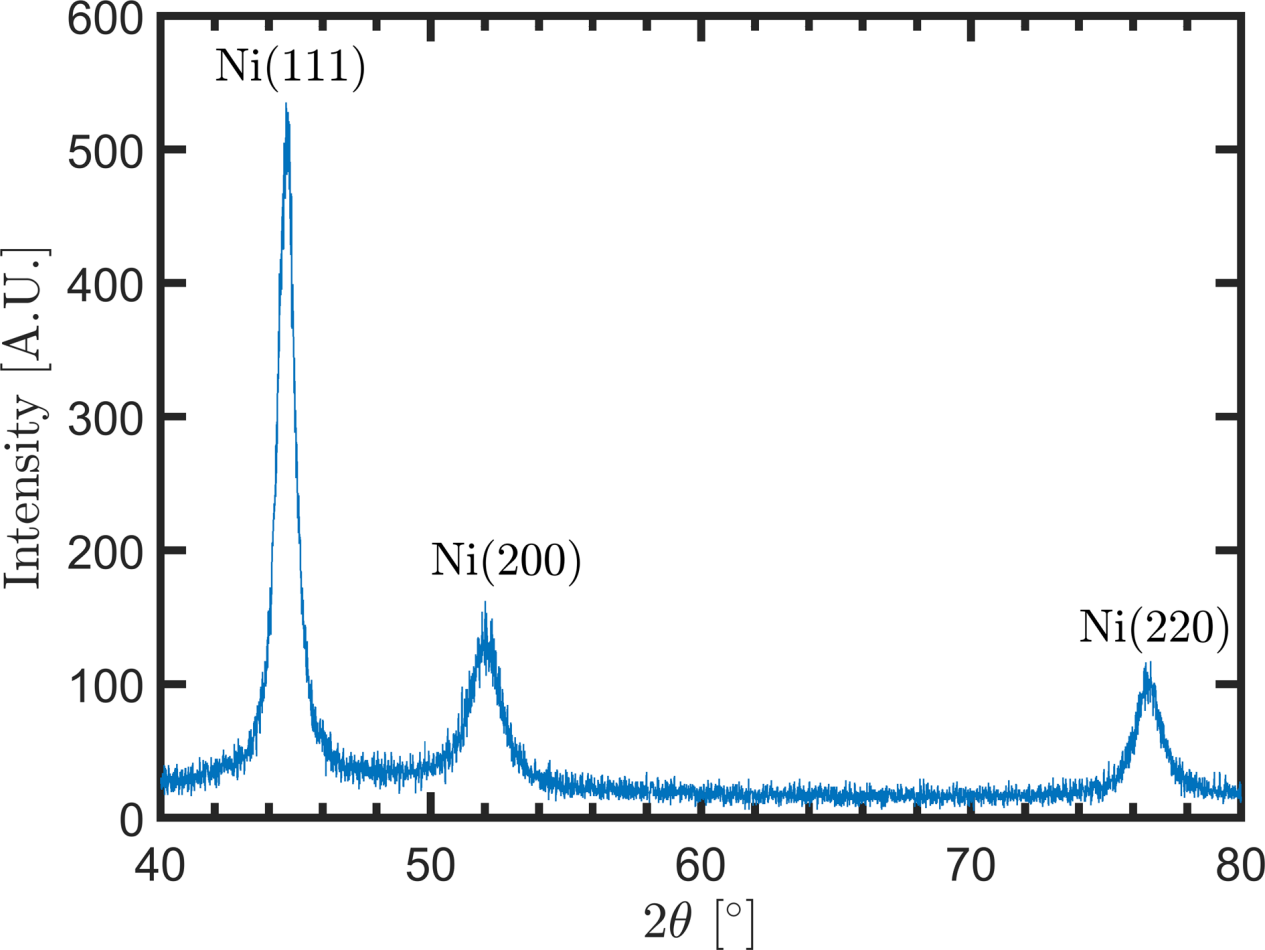
GiXRD pattern of the nickel film deposited by dcMS, conventional position, at 0.6 Pa, and 150 W power.

To investigate the microstructure of our Ni films, GiXRD analysis was carried out. Figure 3 exhibits a GiXRD pattern of a dcMS-deposited Ni film in the conventional position facing the target. The peak at 2θ = 44.5° is dominant in the GiXRD pattern. This peak has been assigned to fcc Ni(111). The peak at 2θ = 51.8° is assigned to fcc Ni(200) and the peak at 2θ = 76.3° to fcc Ni(220) [ICDD 00-004-0850]. Surprisingly, the method of deposition (HiPIMS and dcMS) and degree of tilt angle do not change the GiXRD pattern (relative peak intensities) of the deposited Ni films. The conventional XRD signal was weak due to the low film thickness (not shown).

Our thickness uniformity measurements show that obliquely deposited HiPIMS films are remarkably more uniform than dcMS-deposited films under the same tilt angle. Table 1 exhibits that depositing by HiPIMS results in 69% (at 35°) and 42% (at 70°) more uniform films than dcMS in terms of thickness. Our results agree with the recent findings of Keraudy et al. [53] that HiPIMS-deposited Ni films are denser, better crystallized and exhibit better uniformity than dcMS-deposited films, while the dcMS deposition rate is roughly twice the HiPIMS rate.

**Table 1:** Thickness uniformity of the nickel films deposited under various tilt angles and deposition methods. *d*_ave_ is the average film thickness measured by XRR. Δ*d* is the thickness difference across the deposited film along the direction of the tilt angle.

method	tilt angle	*d*_ave_	Δ*d*	Δ*d*/*d*_ave_
	[°]	[nm]	[nm]	[%]

HiPIMS	35	52	2.6	5
dcMS	35	50	8	16
HiPIMS	70	43	6	14
dcMS	70	50	12	24

**Figure 4:**
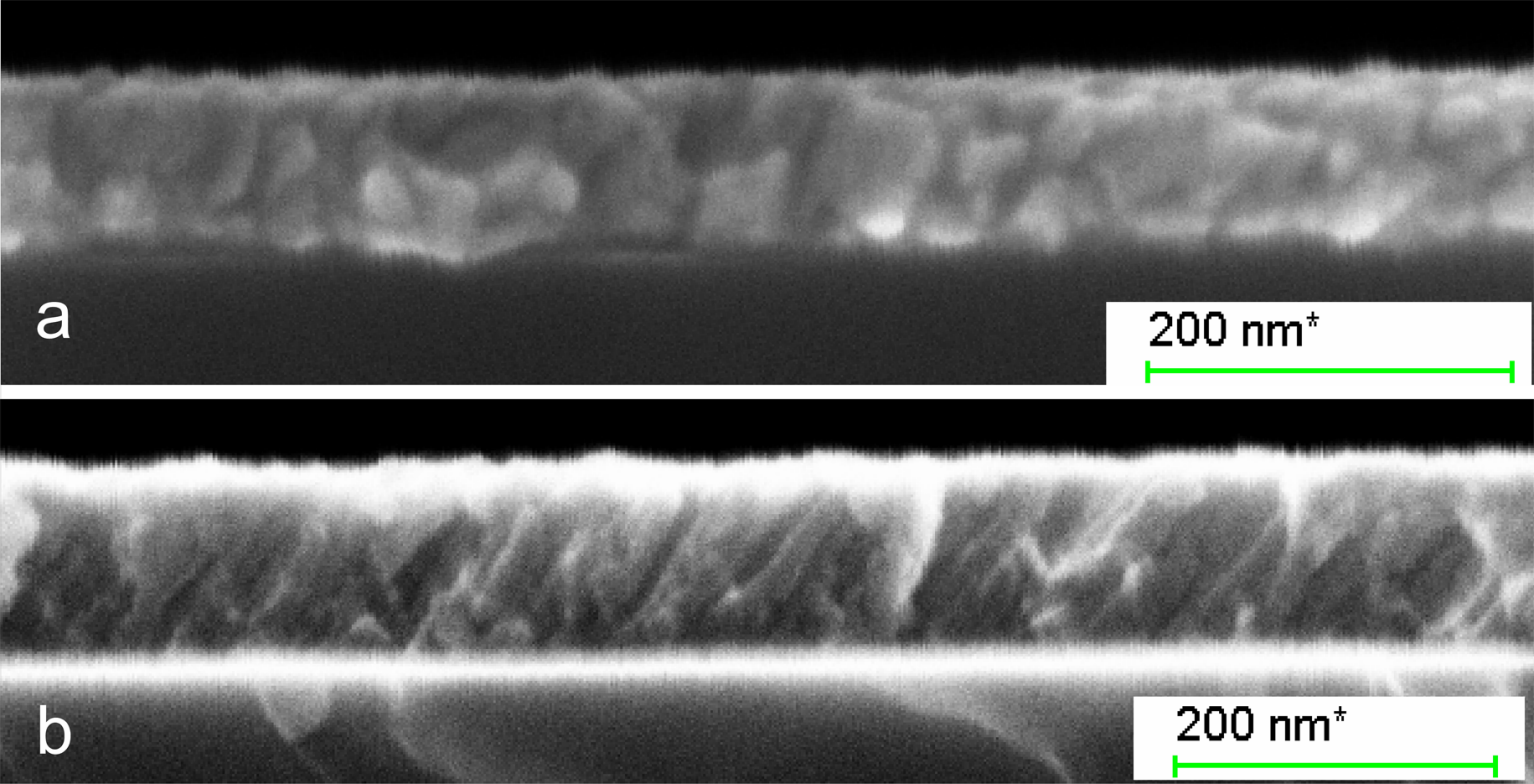
Cross-sectional SEM image of nickel films that were deposited by (a) HiPIMS, and (b) dcMS at 70° substrate tilt angle, at 0.6 Pa, and 150 W power.

Figure 4 depicts cross-sectional SEM images of HiPIMS- and dcMS-deposited films under 70° substrate tilt angle. The dcMS-deposited film exhibits inclined columnar growth with the column length extending through the entire film thickness. In contrast, the HiPIMS-deposited film shows grains that are smaller than the film thickness. The columnar grains of the dcMS-deposited film are grown with 32° incline on the substrate while the HiPIMS film grains do not show a well-defined inclined growth, although some grains are elongated toward the incoming flux.

The angle between columnar grains and substrate normal (β) is different and generally smaller than the angle between the substrate normal and the target (α). Both experimental results [38] and simulations [54] agree on the relation

[1] 2tanβ=tanα.

According to the Equation 1, β is expected to be around 54° for deposition under 70° tilt angle, while it was measured to be roughly 32° for dcMS-deposited film. This is probably because the abovementioned studies consider a small PVD target and low pressure (collision-free) conditions analogous with electron beam and thermal evaporation methods. However, at our working gas pressure the mean free path is around 11 mm which is remarkably shorter than target to substrate distance (250 mm). Besides, Elofsson et al. [55] show that the melting point of the deposited materials impacts the inclined growth of columns by affecting their surface diffusion. Thus, a variation in β is expected for depositing materials with different melting point.

The less tilted grains and the higher thickness uniformity of HiPIMS-deposited films can be explained by a different distribution angle of incoming flux to the substrate in those discharges. There have been a few investigations on this matter that all agree that the magnetic field plays a significant role in the profile of deposition. We have recently shown that, depending on the stationary magnetic field configuration, HiPIMS deposition may result in a more uniform film thickness than dcMS deposition [31]. Furthermore, Qiu et al. [56] showed that the target voltage, magnetic field strength and geometry can affect the shape of the racetrack and the target utilization. Indeed, in a HiPIMS discharge a wider current distribution on the target is expected due to the remarkably higher discharge current and cathode voltage [57]. In other words, the racetrack area could be wider during HiPIMS operation, which, in turn, can lead to a broader profile of sputtered material in terms of directionality. Furthermore, a potential difference of 1–5 V is expected between the plasma and the grounded substrate [58]. In the presence of highly ionized sputtered materials produced by HiPIMS discharge, this potential difference accelerates the ionized flux toward the substrate normal across the sheath and results in a better thickness uniformity as well as less inclined grain growth [58]. In addition, in the HiPIMS process, energetic ions are likely to have enough kinetic energy to induce some mobility of the film forming species on the film surface, which eliminates the columnar growth caused by the shadow effect. Greczynki et al. [42] and Elofsson et al. [55] have studied the HiPIMS growth of metal films on a tilted substrate as a function of peak discharge current density *J*_D_*_,_*_peak_. They showed that for a higher *J*_D_*_,_*_peak_, and thereby a larger degree of ionization of the sputtered material, a smaller tilt angle of the columnar microstructure is observed, i.e., the columns grow closer to the substrate normal. Thus, for a highly ionized flux fraction of the sputtered species the effects of the line-of-sight deposition are effectively eliminated and the film growth proceeds more or less unaffected by the substrate tilt. They have also experimentally rejected the role of deposition rate on the tilted growth of grains. Furthermore, Alami et al. [59] demonstrated that deposition using a peak current density *J*_D_*_,_*_peak_ = 1 A/cm^2^ (close to our *J*_D_*_,_*_peak_ = 0.77 A/cm^2^) results in film densification and suppression of the columnar structure, and columns start to grow on existing columns or repeated nucleation occurs. As the peak discharge current density was increased further to *J*_D_*_,_*_peak_ = 4 A/cm^2^ they observed that a film with a featureless morphology developed.

Smaller grain sizes in HiPIMS-deposited films than in dcMS-deposited films have been previously reported [60–61]. They originate from the bombardment of the film surface by energetic ions during deposition, which constantly creates new sites for growing new crystallites. This, in consequence, leads to smaller grain sizes [62–63].

### Magnetic properties

**Figure 5:**
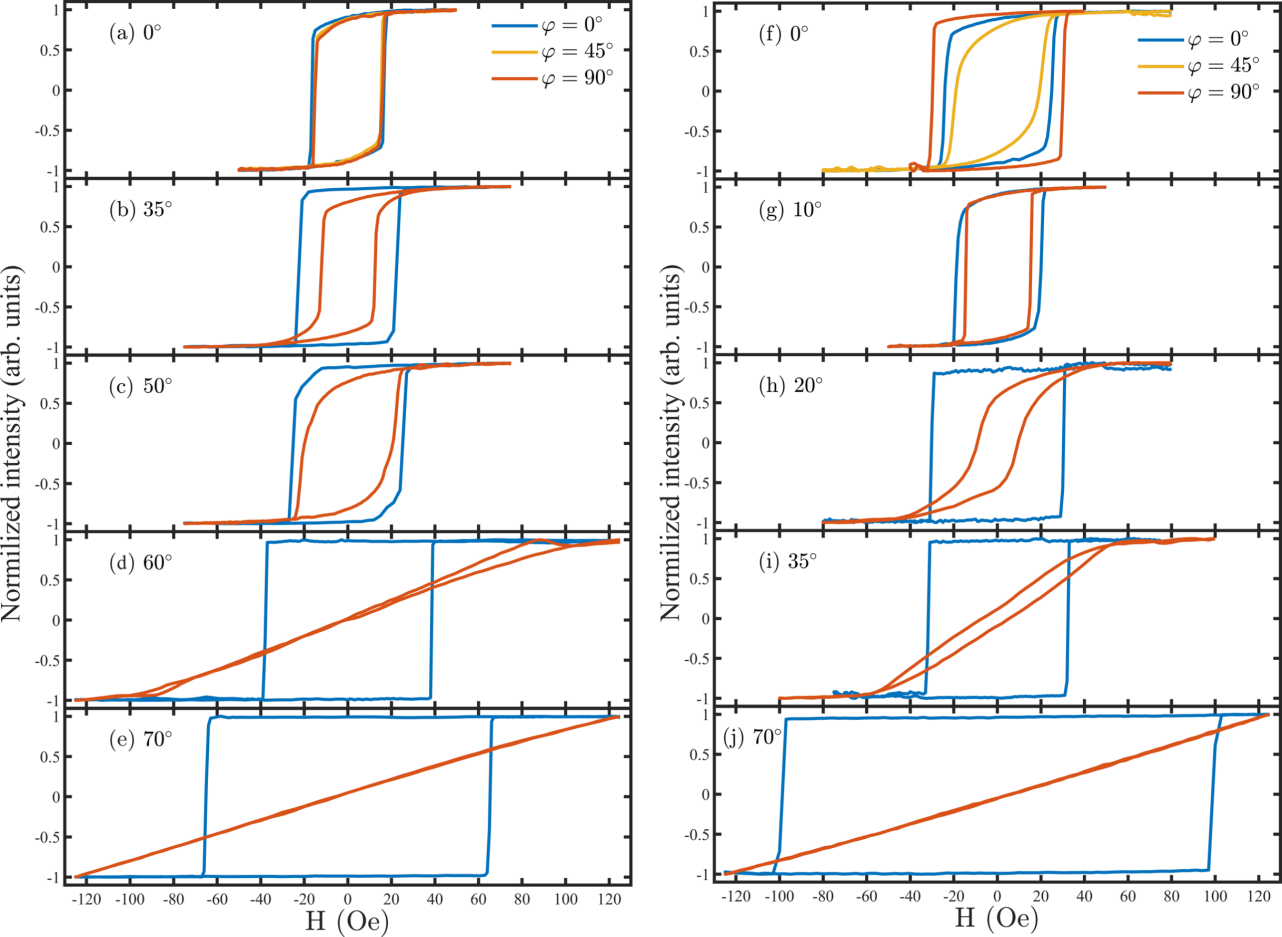
MOKE loops of nickel films that were deposited by (a–e) HiPIMS and (f–j) dcMS, at various tilt angles ranging from 0° to 70°, at 0.6 Pa working gas pressure, and 150 W average power. Each figure shows the in-plane angle of the applied magnetic field with respect to the incoming flux direction.

We used MOKE to explore the magnetic properties of the nickel films. The results are shown in Figure 5 for HiPIMS and dcMS-deposited films. The films deposited by HiPIMS at 0, 35° and 50° tilt angles are more or less magnetically isotropic in-plane. However, the films deposited under 60° and 70° present uniaxial behavior, i.e., a linear hard axis along the angle of incoming sputtered flux and a square easy axis perpendicular to that in the plane. The films deposited by dcMS at tilt angles of 0°, 10° and 20° also show more or less isotropic behavior. Further increasing the tilt angle leads to a uniaxial anisotropy in dcMS-deposited films at tilt angles of 35° (Figure 5i) and 70° (Figure 5e).

Thus, for both deposition methods there is an intermediate tilt angle (ca. 50° for HiPIMS and ca. 20° for dcMS) at which the films present hysteresis loops with different values of *H*_c_ when magnetic field is applied parallel and perpendicular to the incoming flux direction. To determine the window in which a transition occurs from isotropic to uniaxial anisotropy is important for practical purposes. For instance, in the films deposited at these intermediate tilt angles the *H*_c_ value of the loops is different and the loop exhibiting lower *H*_c_ values is more rounded. The latter loop is perpendicular to the angle of incidence and it becomes a hard axis at larger tilt angles.

**Figure 6:**
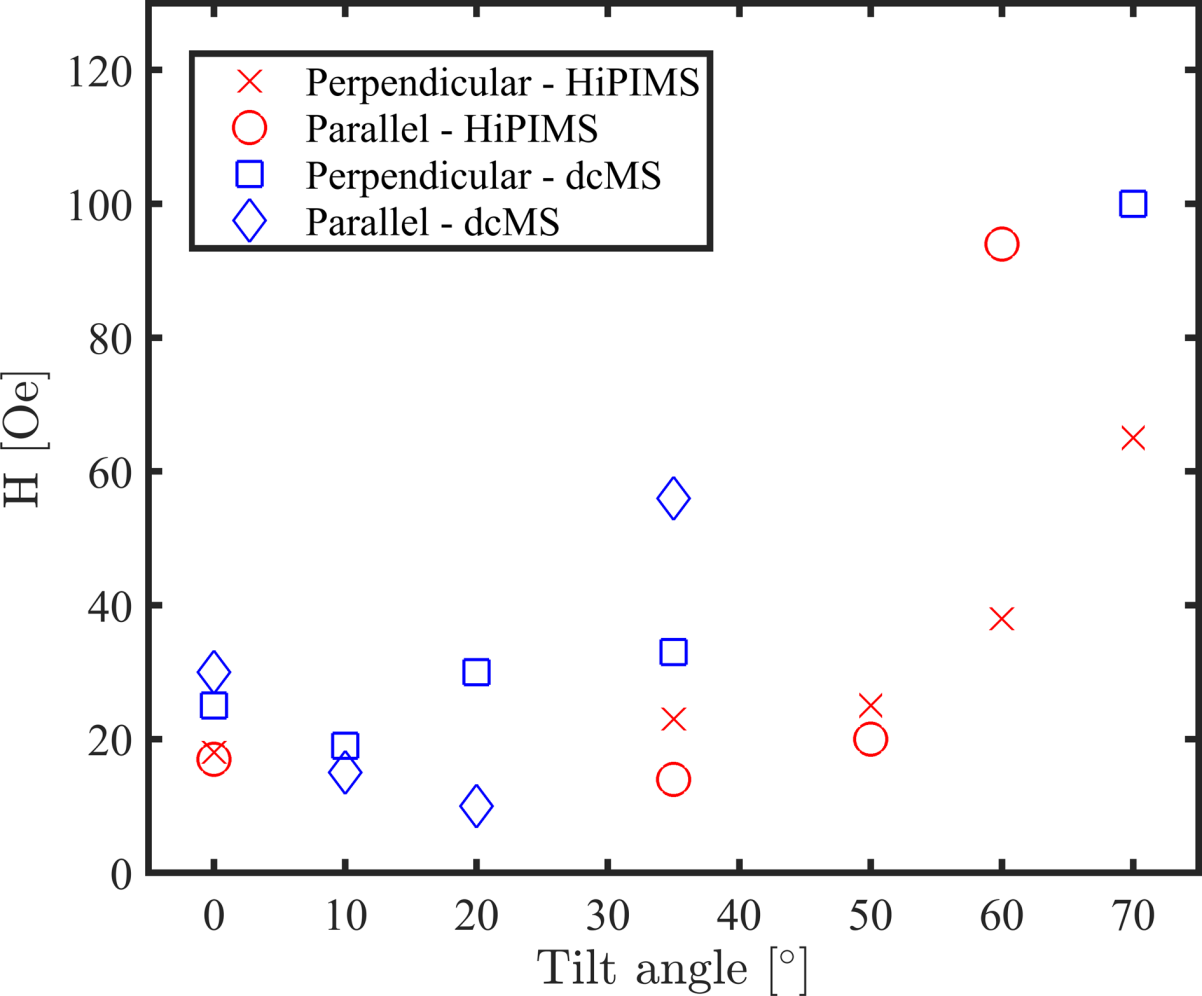
The *H*_c_ and *H*_k_ (for samples with uniaxial anisotropy) of our nickel films measured using MOKE with the magnetic field applied parallel and perpendicular to the sputtered flux direction, respectively. Uniaxial anisotropy is presented in HiPIMS samples of 60° and 70°, and in dcMS of 35° and 70°. The *H*_k_ of films deposited at 70° was out of the measurement range.

The coercivity and anisotropy fields of our Ni films are plotted as a function of the tilt angle in Figure 6. It is worth mentioning that regardless of the type of anisotropy, along the easy direction of magnetization, *H*_c_ of the HiPIMS-deposited films increases with increasing tilt angle. This is also true for *H*_k_ for the samples with uniaxial anisotropy. In contrast to the HiPIMS results, dcMS-deposited films present similar *H*_c_ values with increasing tilt angle up to 35° and show an increase with further increase in tilt angle. For deposition at 70° tilt angle, the anisotropy field of both dcMS- and HiPIMS-deposited samples were higher than the measurement range in our MOKE setup.

Interestingly, HiPIMS-deposited Ni films are magnetically softer than dcMS-deposited films at the same tilt angle. We believe that the smaller grain size of HiPIMS-deposited films (shown in Figure 4) is the main reason for soft magnetism of the films. Poolcharuansin et al. [64] have shown that Ni thin film deposition using an inverted gapped-target sputter magnetron results in smaller grain size and consequently magnetically softer films than dcMS-deposited films.

To summarize, transition from isotropic to uniaxial anisotropy occurs above 50° tilt angle for HiPIMS deposition while it is around 35° in dcMS. It is probably due to less inclined columnar growth in HiPIMS-deposited films as is shown in Figure 4. We have studied the in-plane magnetic properties of deposited films using VSM and the results are in agreement with the MOKE study (not shown here).
